# Homoconjugation *vs.* Exciton Coupling in Chiral α,β-Unsaturated Bicyclo[3.3.1]nonane Dinitrile and Carboxylic Acids

**DOI:** 10.3390/molecules19079893

**Published:** 2014-07-08

**Authors:** Gintautas Bagdžiūnas, Eugenijus Butkus, Sigitas Stončius

**Affiliations:** 1 Department of Organic Chemistry, Vilnius University, Naugarduko 24, 03225 Vilnius, Lithuania; E-Mails: gintautas.bagdziunas@ktu.lt (G.B.); eugenijus.butkus@chf.vu.lt (E.B.); 2 Present address: Department of Polymer Chemistry and Technology, Kaunas University of Technology, Radvilėnų pl. 19, 50254 Kaunas, Lithuania

**Keywords:** circular dichroism, conformational analysis, TD-DFT calculations, transannular interactions, homoconjugation

## Abstract

The chiroptical properties of enantiomerically pure bicyclo[3.3.1]nona-2,6-diene-2,6-dicarbonitrile and related acids were studied by circular dichroism spectroscopy and theoretical computations. A consideration of the molecular structure of the synthesized difunctional compounds revealed that chromophores are predisposed to transannular through-space interaction due to a favourable conformation of the bicyclic skeleton and a rather small interchromophoric distance. Evidence for non-exciton-type coupling between the two acrylonitrile and acrylate moieties in **3** and **4**, respectively, was obtained by chiroptical spectroscopy and DFT calculations.

## 1. Introduction

Cyclic α,β-unsaturated carboxylic acids are found in various chiral natural products such as the heteroyohimbine alkaloids [1] or shikimic and chorismic acids, which are involved in biosynthetic processes and used in enzyme action studies [2]. The latter acids are precursors for the synthesis of biologically active compounds [3,4] and are important biochemical intermediates [5]. Chiroptical methods and, in particular, circular dichroism (CD) spectroscopy are nowadays well-established techniques for the determination of the absolute stereochemistry of chiral compounds. A variety of methodologies, ranging from empirical rules to *ab initio* quantum mechanical methods, have been devised for the correlation of the chiroptical properties with the absolute configuration [6]. In particular, rapid development of high-level quantum mechanical methods led to the use of advanced calculations, not only for the absolute stereochemistry determination of model, relatively small compounds, but also for studies of conformational changes and chromophore interactions in large biochemically relevant structures, such as proteins [7]. Nevertheless, the question of setting the absolute configuration of polyfunctional molecules remains a nontrivial task and may become a complicated issue in cyclic α,β-unsaturated carboxy compounds [8] due to the complex character of the chromophore. In general, the application of electronic circular dichroism measurements led to the correlation of the sign and magnitude of the observed Cotton (CE) effect with the absolute configuration of chiral molecules containing various chromophores [9,10]. In particular, the related α,β-unsaturated ketone chromophore belongs to the group of chromophores that have been investigated both experimentally and theoretically over the last years [11]. A number of various sector and/or helicity rules have been proposed to correlate the sign of the CEs that is observed in the spectral region between 200 and 350 nm with the absolute configuration of α,β-enone molecules [12]. These rules are derived from the observation that the CD and UV spectra of enones are affected by substituents located in the proximity of this chromophore altering its electronic structure as well as spatial dimension [13].

Thus, suitable model structures with several chromophores are of considerable interest for studying chiroptical properties in more detail. Our attention turned to acrylonitrile and acrylate chromophores in the chiral bicyclo[3.3.1]nonane framework that has been shown to be a proper conformationally diverse molecular structure for stereochemical studies [14,15,16]. In addition, these chromophores have received little attention over last years.

In this work the synthesis of bicyclo[3.3.1]nonane α,β-unsaturated dinitrile and the corresponding acids was accomplished. The molecular conformation of unsaturated derivatives was investigated through a combination of molecular mechanics and DFT calculations. Enantiomerically pure compounds of this framework were employed as suitable models to study chiroptical properties, *i.e.*, the effects of electronic interactions in the generation of rotational strength in the electronic transitions and conceivable chromophore interaction by CD spectroscopy and TD-DFT computations.

## 2. Results and Discussion

**Scheme 1:**
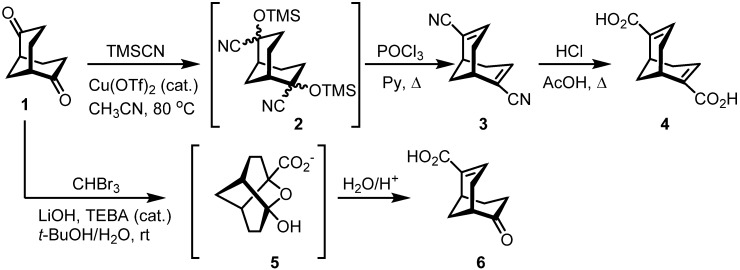
Synthesis of unsaturated derivatives **3**, **4**, **6**.

Difunctional α,β-unsaturated nitrile **3** and acid **4** (Scheme 1) were prepared using the typical synthetic methodology, which involves sequential dehydration of ketone cyanohydrins using phosphorus oxychloride/pyridine or thionyl chloride, followed by acidic or alkaline hydrolysis of the resulting conjugated nitrile. Quast *et al.* [17] used this method for the synthesis of bicyclo[3.3.1]nona-2,6-diene-2,6-dicarbonitrile and 2,6-dicyanobarbaralanes from the racemic bicyclo[3.3.1]nonane-2,6-dione **1**. Herein we report on the synthesis of enantiomerically pure dinitrile **3** from (+)-(*1S,5S*)-bicyclo[3.3.1]nonane-2,6-dione (**1**) (Scheme 1).

We found that the Cu(OTf)_2_ is an effective catalyst [18] for nucleophilic addition of TMSCN to diketone **1**. The cyanohydrin **2** was dehydrated using phosphorus oxychloride and pyridine and the so formed dinitrile **3** was hydrolyzed with concentrated HCl in acetic acid to yield corresponding α,β-unsaturated acid **4**. An attempt to synthesize diacid **4** via an addition of bromoform to ketone **1** under alkaline conditions [19] was also made. Interestingly, only unsaturated keto carboxylic acid **6** was isolated, whose structure was confirmed by its ^13^C-NMR spectrum in which characteristic ketone and carboxyl group carbonyl signals were observed at 213.9 and 171.5 ppm, respectively. It is likely that the reaction takes place through tricyclic oxatwistane intermediate **5**, formation of which precludes the nucleophilic addition of the Br_3_C^−^ nucleophile to the second carbonyl group to give difunctional derivative **4**. Subsequently, the oxatricyclic intermediate **5** was easily hydrolyzed under acidic conditions to afford **6** (Scheme 1). A compound similar to **5** was isolated and identified by Quast in the reaction of **1** with trimethyl(trifluoromethyl)silane [20].

Conformational analysis of dinitrile **3**, diacid **4** and monoacid **6** was performed with SPARTAN 10 using the Monte-Carlo method and MMFF94 force field. The minimum energy conformers found by molecular mechanics have been further optimized at the DFT/B3LYP/aug-cc-pVDZ level, followed by calculations of their harmonic vibrational frequencies to verify their stability and to calculate conformational free energies (Table 1). 

**Table 1:** .Relative free energies, populations, symmetries, and torsion angles for low energy conformers of **4** and **6**.

Compound	Conformer ^a^	ΔG, kcal/mol	Distribution,%	Angle (C=C-C=O), deg. (Symmetry)
**4**	*trans-trans*	0.00	69.4	178.6 ( *C_2_*)
	*cis-trans*	0.53	28.0	−0.3, 178.6 ( *C_1_*)
	*cis-cis*	1.93	2.6	−0.9 ( *C_2_*)
**6**	*trans-chair*	0.00	77.7	179.5 ( *C_1_*)
	*cis-chair*	0.78	20.4	−0.6 ( *C_1_*)
	*trans-boat*	2.26	1.6	−179.4 ( *C_1_*)
	*cis*-*boat*	3.14	0.3	0 ( *C_1_*)

^a^ chair/boat denotes the conformation of the cyclohexanone ring.

**Figure 1:**
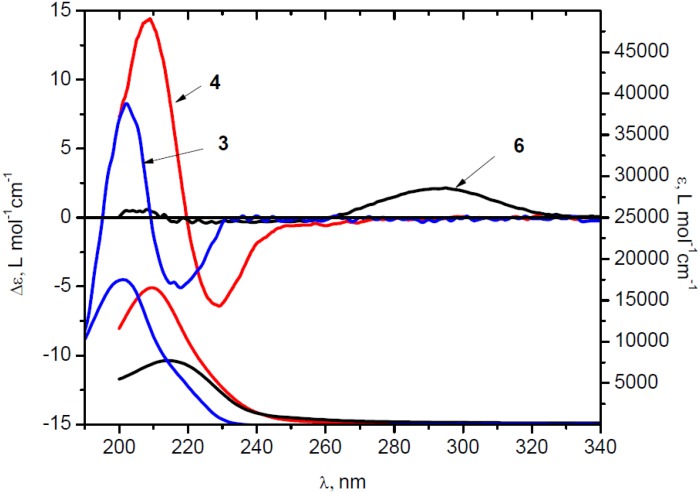
CD and UV spectra of dinitrile (+)*-***3** ( 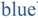) in MeCN, diacid (+)*-***4** (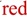) and ketoacid(+)*-***6** (solid) in EtOH.

Conformational flexibility of the unsaturated bicyclic framework is notably reduced and for difunctional compounds **3** and **4** the double half-chair conformation was found to be the most stable. In the case of **6**, a half-chair/chair conformation of the bicyclic framework was found to be the most stable and prevailed over other conformations (Table 1). However, for compounds **4** and **6** the carboxyl group could be either s-*cis* or s-*trans* with respect to the C=C─C=O bond, thus yielding three and four conformers of **4** and **6**, respectively. The conformational analysis of dinitrile **3** gave only one stable conformer. Chiroptical properties of chiral α,β-unsaturated nitrile **3** and carboxylic acids **4** and **6** were also investigated. The experimental CD and UV spectra are shown in Figure 1. 

**Figure 2:**
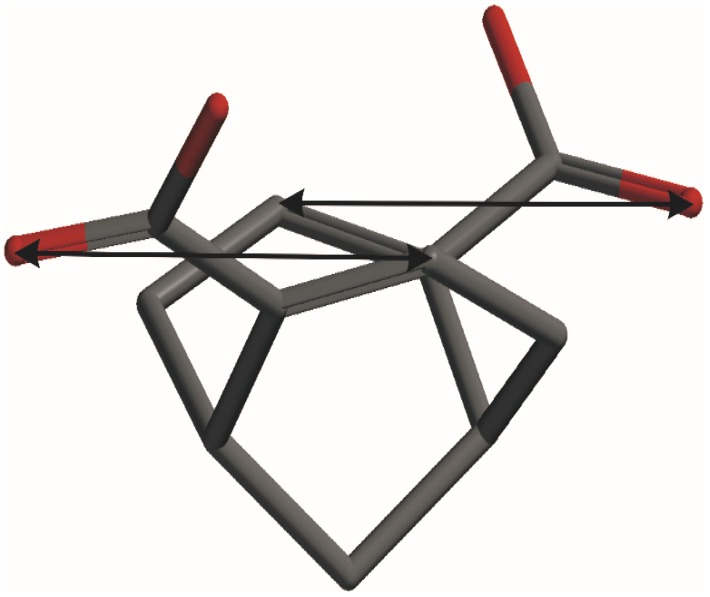
Orientation of the two long-axis polarized π→π* transition dipoles in **4**.

The positive CE around 290 nm in the CD spectrum of **6** is assigned to n→π* transition of the carbonyl group and the intensity is typical for this chromophore. The sign of this CE can be accurately predicted using the octant rule for the carbonyl chromophore. No CE’s associated with the π→π* transitions of the α,β-unsaturated carboxylate moiety around 200–240 nm were observed in the experimental CD of **6**, suggesting negligible (canceling) external perturbation of virtually planar (non-skewed) chromophore (Table 1) by dissymetrically disposed substituents. By contrast, the interpretation of the experimental CD spectra of **3** and **4**, and the assignment of observed CE’s to particular electronic transitions is more complicated because the strong bisignate absorption band is not due to the exciton-type coupling [21]. The orientation of the two unsaturated chromofores in these dichromophoric molecules is close to parallel and the π→π* transition electric dipoles do not form a chiral array prerequisite for exciton coupling [22], as illustrated for the most stable *trans-trans* conformer of diacid **4** (Figure 2). In addition, the corresponding absorption band in the UV spectrum of **4** is blue-shifted in comparison with the monounsaturated congener **6**, as can be expected in the case of stacked chromophores with parallel orientation of the two relevant transition dipoles [23].

Nevertheless, the bisignate CE in the CD spectra apparently originates from the coupling between unsaturated fragments, e.g., in diacid **4**, because the signal around 200–240 nm is not observed in the case of monounsaturated derivative **6**. Linhard *et al.* assigned the CD spectra bands around 230 nm of related α-alkoxyacrylates to π→π* transition and related the sign of CE with the intrinsic chirality of the unsaturated moiety, reflected by the twist of carbonyl of carboxylate group in relation to C=C bond [24]. In the case of **3** and **4**, however, the sign of the observed CE’s in the CD spectra (Figure 1) is not correlated with the twisting of the conjugated chromophores, which are essentially planar as suggested by DFT calculations (Table 1).

**Figure 3:**
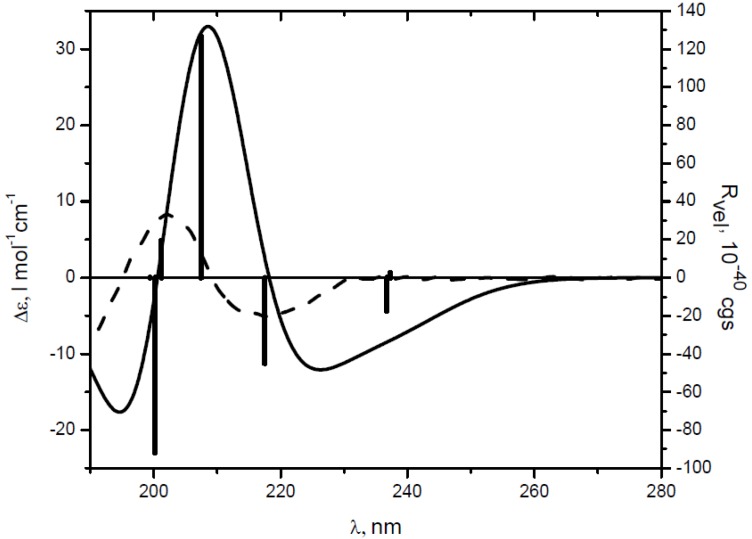
Comparison of the calculated (solid) and experimental (- - ) CD spectra of dinitrile **3** at the B3LYP/aug-cc-pVDZ level (σ = 0.30 eV); bars represent the rotatory strength.

We calculated the CD spectra of dinitrile *(1S,5S)-***3** and diacid *(1S,5S)-***4** with the TD-DFT/B3LYP/aug-cc-pVDZ method and performed analysis of the molecular orbitals taking into account only intense rotatory strengths (Table 2). Thus, the negative CE band at 218 nm in CD spectra of **3** is congruous with theoretical transition at 217 nm with −44.91 (in 10^−4^^0^ cgs units) rotational strength (Figure 3). 

**Table 2:** Properties of selected transitions and their contribution to the CD spectra of dinitrile **3** and diacid **4** (*trans-trans* conformer) calculated at the B3LYP/aug-cc-pVDZ level*.*

Comp.	λ, nm (Δε) ^a^	Transition	λ, nm	*f* ^b^	R_vel_^c^	Contributions ^d^
**3**	218 (−5.16)	3	217.5	0.0015	−44.91	45–47 (61%), 44–46 (34%)
	202 (8.46)	4	207.5	0.501	126.42	44–47 (48%), 45–46 (33%)
**4**	229 (−6.41)	5	226.2	0.0019	−50.62	54–57 (66%), 55–56 (29%),
	209 (14.4)	8	215.2	0.530	74.32	55–57 (45%), 54–56 (45%)

^a^ experimental CD; ^b^ oscillator strength; ^c^ Rotatory strength in velocity form (10^−4^^0^ cgs); ^d^ contributions more than 5% are selected.

**Figure 4:**
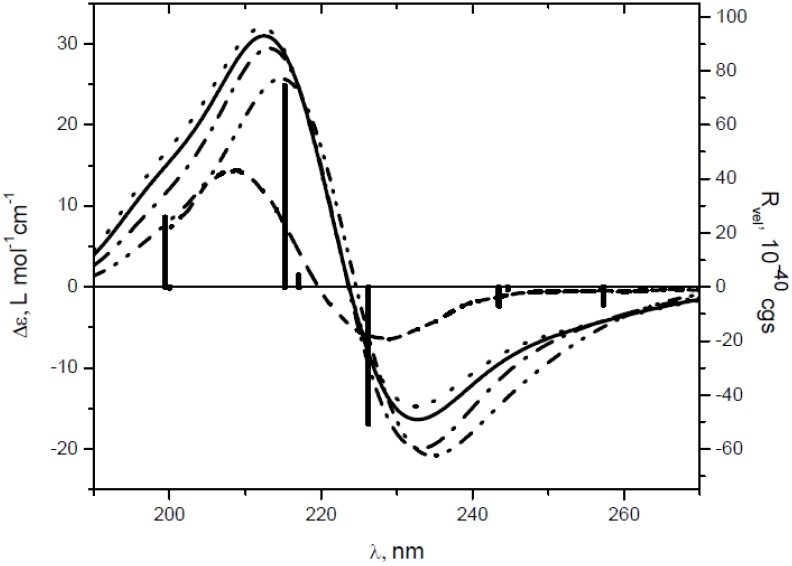
Comparison of experimental (- - ) and theoretical spectra of diacid **4**, calculated at the B3LYP/aug-cc-pVDZ level (σ = 0.30 eV) for individual conformers (*trans-trans* (∙∙∙∙), *cis-trans* (-∙-), *cis-cis* (-∙∙-)), and Boltzmann-averaged (solid) CD spectrum; bars represent the rotatory strength for the most abundant *trans-trans* conformer.

Accordingly, single-electron excitation at 226 nm with similar rotatory strength (−50.62 × 10^−4^^0^ cgs) for prevailing *trans-trans* conformer of diacid **4** was predicted (Figure 4 and Table 2). The positive CE’s in CD spectra of **3** and **4** at 202 and 209 nm could be attributed to the calculated excitations at 207 nm (126.42 × 10^−4^^0^ cgs) and 215 nm (74.32 × 10^−4^^0^ cgs), respectively. The predicted spectra for the minor *cis-trans* and *cis-cis* conformers of **4** were very similar to that of *trans-trans-***4** regarding the pattern, absolute and relative intensity of the Cotton effects (Figure 4).

To address the possible solvent effect on the conformational equilibrium and calculated chiroptical properties, dinitrile **3** and conformers of diacid **4** were reoptimized using the polarizable continuum model (PCM) in conjunction with B3LYP/aug-cc-pVDZ, followed by calculation of their harmonic vibrational frequencies and CD spectra at the same level of theory. Apart from a slight bathochromic shift (ca 2 nm) and notably (*ca*. 20%) increased CD intensity, the calculated CD spectra for dinitrile **3** in the gas phase and acetonitrile solution were virtually identical. In the case of **4**, the geometry of the respective conformers reoptimized using PCM were similar to those obtained in the gas phase, but the relative stability of *cis-trans*- and *cis-cis*-conformers, characterized by a substantial dipole moment, increased in ethanol solution. Population of *trans-trans-*, *cis-trans-* and *cis-cis*-**4 ** using PCM was calculated to be 51.8%, 41.7% and 6.5%, respectively (cf. Table 1). Comparison of the Boltzmann-averaged CD spectra calculated for **4** in the gas phase and ethanol solution, however, revealed no major differences. As in the case of **3**, CD spectrum of **4** predicted using PCM was bathochromically shifted by ca 6 nm, whereas the intensity of the CEs decreased by ca 20%, e.g., the respective transitions for *trans-trans-***4** conformer were predicted at 229.0 and 221.1 nm with R_vel_ −39.79 × 10^−4^^0^ and 57.17 × 10^−4^^0^, respectively (cf. Table 2).

**Figure 5:**
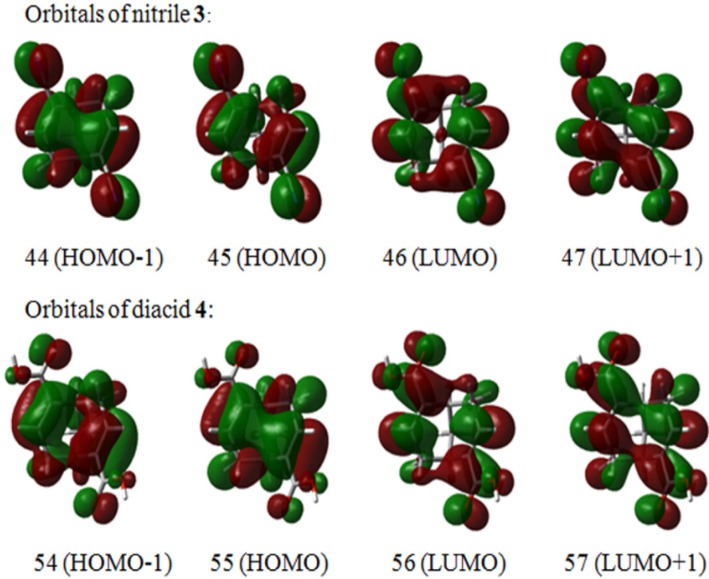
Molecular orbitals associated with the main transitions of dinitrile **3** and diacid **4**.

The calculated transitions correspond to π→π* excitations of the unsaturated chromophores and the relevant frontier molecular orbitals (MO) are shown in Figure 5. MO analysis suggests a substantial through-space interaction [25,26] of π orbitals at the *endo* (concave) face in dichromophoric compounds **3** and **4**. Bicyclic frameworks were shown to be particularly well suited for the studies of electron delocalization through homoconjugation in terms of direct (“through-bond”) and indirect (“through-space”) interactions from localized sets of orbitals or chromophores. Such interaction of π–electron systems in norbornadienes [27], norbornenones [28], barrelenes [29], bicyclo[3.3.1]nona-dienones [30] and related frameworks [31] was evidenced by photoelectron, NMR and CD spectroscopy. In the case of **3** and **4**, two localized (isolated) chromophores are predisposed to transannular through-space interaction due to a favourable conformation of bicyclic skeleton and a rather small interchromophoric distance (ca. 3.2–3.3 Å, measured as C_2_–C_6_ and C_2_–C_7_ distance). 

An in-phase (symmetric) linear combinations of the corresponding MO’s results in π-extended (homoconjugated) molecular orbitals, *i.e.*, 44 (HOMO-1), 47 (LUMO+1) and 55 (HOMO), 57 (LUMO+1) for **3** and **4**, respectively. The negative CE in the calculated CD spectra of **3** and **4** at 218–229 nm originates from two single-electron transitions, involving excitations from “localized” to homoconjugated (MO(45)→(MO)47, MO(54)→MO(57)) and excitations from homoconjugated to “localized” (MO(44)→(MO)46, MO(55)→MO(56)) molecular orbitals (Table 2, Figure 5). The calculated positive rotational strength at 202 and 209 nm for **3** and **4**, respectively, are described as mixtures of excitations involving homoconjugated molecular orbitals, that is 44 (HOMO-1)→47 (LUMO+1) and 55 (HOMO)→57 (LUMO+1) (Table 2), and excitations form “localized” occupied MO to “localized” LUMO, *i.e.*, MO(45)→(MO)46 and MO(54)→MO(56) for **3** and **4**, respectively. HOMO and LUMO of **3** and **4** should be viewed as delocalized molecular orbitals, where a clear electronic communication between the π (and π*) orbitals of each unsaturated chromophore exists. That is to say the present system should be treated as a single conjugated electron system where the two unsaturated moieties are conjugated to each other, losing their nature as an independent unsaturated chromophore. As a result, the analysis of stereostructure of such fully delocalized conjugated monochromophores by the exciton chirality method is not justified [32]. The extended molecular orbitals resemble the π-molecular orbitals of conjugated transoid dienes [33] and, consequently, the observed/calculated sign of the CE’s is related to the inherent dissymmetry (helicity) of the extended unsaturated monochromophore [34,35]. The latter is *C_2_*-symmetric and acquires left-handed (M) helicity, roughly expressed as a C_2_–C_1_---C_5_–C_6_ dihedral angle (ca. –100 degrees), in *(1S,5S)-***3** and *(1S,5S)-***4**. In inherently dissymmetric (helical) *C_2_*-symmetric chromophores the electronic excitations can be classified as A or B type, according to their orientation with respect to the two-fold symmetry axis. The bisignate CD in the case of **3** and **4**, negative at the longer wavelengths and positive at shorter wavelengths, is in accordance with a general helicity rule [36,37,38,39], which predicts a negative rotational strength for the transition of lowest energy, polarized perpendicular to the *C*_2_ axis in a chromophore of M helicity; positive rotational strength is predicted for the higher energy transition, which is polarized parallel to the symmetry axis of the chromophore.

## 3. Experimental Section

### 3.1. General Information

Melting points were determined with a Gallenkamp melting apparatus in capillary tubes and are not corrected. IR spectra were recorded in KBr pellets on a Perkin–Elmer Spectrum BX spectrometer (Waltham, MA, USA). Proton (300 MHz) and carbon (75 MHz) nuclear magnetic resonance spectra were recorded on a Varian Inova 300 spectrometer and chemical shifts are reported in parts per million relative to solvent resonance signal as an internal standard. The CD and UV spectra were recorded with a JASCO J-815 spectrometer (Tokyo, Japan) at 20 °C in a 0.10 cm cell with scaning rate of 50 nm/min. The stock solutions were prepared by weighting compound into volumetric flask and diluting with UV-grade solvents. The CD spectra were measured in millidegrees and normalized into Δ*ε*_max_ [L mol^−1^ cm^−1^]/λ[nm] units. Optical rotations were measured on a KRÜSS P3001RS automatic digital polarimeter (Hamburg, Germany) at 589 nm and [α]^20^ values are given in 10^−1^ deg cm^2^ g^−1^, and concentrations are given in units of g/100 cm^3^. Elemental analyses were performed by the analytical laboratory of the Chemistry Faculty, Vilnius University. Thin-layer chromatography was carried out on Kieselgel 60 F254 (Merck, Darmstadt, Germany) plates coated with silica gel, and Kieselgel 60 silica gel (0.040–0.063 mm, Merck) was used for column chromatography. The synthesis of starting enantiomerically pure (+)-(*1S,5S*)-bicyclo[3.3.1]nonane-2,6-dione **1** was performed following the procedure described earlier [40].

### 3.2. Theoretical Calculations

Conformational search of diacid **4** and monoacid **6** was performed with SPARTAN’10 [41] using the Monte–Carlo method and MMFF94 force field. The minimum energy conformers found by molecular mechanics have been further optimized using Gaussian software [42] at DFT/B3LYP/aug-cc-pVDZ level, followed by calculations of their harmonic vibrational frequencies to verify their stability and to calculate conformational free energies; population percentages were calculated using ΔG and applying Boltzmann statistics at T = 293.15 K. The electronic circular dichroism CD calculations were performed using Gaussian software and employing TD-DFT approach with the B3LYP hybrid functional combined with the aug-cc-pVDZ basis set. Additionally, for compounds **3** and **4** the geometry of the conformers in acetonitrile (ε = 35.688) and ethanol (ε = 24.852) solution, respectively, was optimized using PCM model at B3LYP/aug-cc-pVDZ level; CD spectra were calculated at the same level of theory. The rotational strength calculations have been carried out both in velocity (*R*_vel_) and length formalism (*R*_len_); the results in the two formalisms were almost identical. Rotatory strengths are reported in the usual cgs units of 10^−4^^0^ esu cm erg G^−1^, where 1 esu cm erg G^−1^ corresponds to 3.336 × 10^−15 ^ C m J T^−1^ in SI units. The CD spectra of the individual conformers were simulated by overlapping Gaussian functions for each transition using SpecDis version 1.61 [43]. The half-width at 1/e of the peak maximum (σ) value of 0.30 eV and *R*_vel_ were used in this work. The calculated peak maxima were not wavelength corrected.

### 3.3. Preparation of Compounds **3**–**6**

(+)-(*1S,5S*)-Bicyclo[3.3.1]nona-2,6-diene-2,6-dicarbonitrile (**3**). TMSCN (0.82 mL, 6.2 mmol) was added to a solution of **1** (234 mg, 1.54 mmol) and Cu(OTf)_2_ (56 mg, 0.15 mmol) in abs. CH_3_CN (5 mL) under an argon atmosphere. The reaction mixture was stirred at 80 °C temperature for 3 days in a closed vessel. The solvent was then evaporated, the residue was diluted with DCM and washed with dist. H_2_O, brine and dried over Na_2_SO_4_. The solution was evaporated to dryness to yield crude **2** (480 mg, 89%) as an yellow oil, which was used without further purification. POCl_3_ (0.76 mL, 8.2 mmol) and pyridine (1.1 mL, 14 mmol) were added to **2 (**480 mg, 1.4 mmol) under an argon atmosphere. The reaction mixture was refluxed for 4 h, then cooled to room temperature and quenched with ice. The resulting mixture was filtered through a pad of Celite, and the pad was washed with DCM. After separation of the layers, the aqueous phase was extracted with DCM (3 × 20 mL). The organic layers were combined, washed with 0.1 M HCl, sat. NaHCO_3_ and dried over Na_2_SO_4_. The residue was purified by column chromatography (eluent ethyl acetate/petroleum ether, 1:3) to yield **3** (186 mg, 71%) as colorless crystals; mp 147–149 °C (racemate mp 189–190 °C [17]); [α]_D_ = +15.5 (*c* 0.71, CHCl_3_); UV, λ_max_ (logε): 201 (4.24); IR (KBr) ν_max_/cm^−1^ 2919, 2208, 1628, 1433; ^1^H-NMR (CDCl_3_, ppm) *δ* 6.62 (dd, *J* = 5.0, 2.5 Hz, 2H), 2.84–2.67 (m, 2H), 2.55–2.26 (m, 4H), 1.80 (t, *J* = 3.2 Hz, 2H); ^13^C-NMR (CDCl_3_, ppm) *δ* 142.6, 118.6, 116.2, 31.0, 29.0, 26.6; Anal. Calcd for C_11_H_10_N_2_: C 77.62; H 5.92; N 16.46. Found: C 77.69; H 5.94; N 16.15.

(+)-(*1S,5S*)-Bicyclo[3.3.1]nona-2,6-diene-2,6-dicarboxylic acid (**4**). A solution of **3** (150 mg, 0.88 mmol) in a mixture of conc. HCl (4 mL) and acetic acid (3 mL) was refluxed for 6 h. The reaction mixture was then cooled to room temperature and kept in a refrigerator at −15 °C temperature for 2 days. The crystals were filtered and washed with Et_2_O to give colorless solid, which was dried under vacuum; yield 140 mg (76%); (+)-**4** (and *rac*-**4)** mp > 220 °C (dec.); [α]_D_ = +28 (*c* 0.25, CHCl_3_); UV, λ_max_ (logε): 209 (4.22); IR (KBr) ν_max_/cm^−1^ 3074, 2931, 1677, 1636, 1417, 1254, 1243; ^1^H-NMR (CD_3_OD, ppm) *δ* 6.98 (dd, *J* = 5.0, 2.3 Hz, 2H), 2.97 (s, 2H), 2.54–2.43 (m, 2H), 2.24 (dd, *J* = 19.7, 5.0 Hz, 2H), 1.74 (t, *J* = 3.0 Hz, 2H); ^13^C-NMR (CD_3_OD, ppm) *δ* 170.1, 139.8, 134.8, 33.4, 29.2, 27.8; Anal. Calcd for C_11_H_12_O_4_: C 63.45; H 5.81. Found: C 63.64; H 5.91.

(+)-(*1S,5S*)-6-Oxobicyclo[3.3.1]non-2-ene-2-carboxylic acid (**6**). Diketone **1** (170 mg, 1.12 mmol) and TEBA (51 mg, 0.22 mmol) in *t-*BuOH (5 mL) were added to a solution of LiOH∙H_2_O (1.88 g, 44.8 mmol) in H_2_O (5 mL) and CHBr_3_ (0.98 mL, 11 mmol). The reaction mixture was stirred at room temperature for 48 h under an argon atmosphere, then washed with toluene. The aqueous phase was acidified with 0.1 M HCl, stirred for 1 h and then extracted with DCM (3 × 10 mL). The organic phase was dried over Na_2_SO_4_ and evaporated. The residue was purified by column chromatography (eluent ethyl acetate / petroleum ether 1:2) to yield **6** (120 mg, 60%) as colorless crystals; mp 95–97 °C; [α]_D_= +166 (*c* 0.307, CHCl_3_); UV, λ_max_ (logε): 214 (3.89); IR (KBr) ν_max_/cm^−1^ 2939, 1704, 1678, 1636, 1420, 1222; ^1^H-NMR (CDCl_3_, ppm) *δ* 10.4 (bs, 1H), 7.26 (dd, *J* = 4.2, 2.7 Hz, 1H), 3.02 (s, 1H), 2.74 (s, 1H), 2.62 (ddd, *J* = 21, 6.9, 2.7 Hz, 1H), 2.42–1.79 (m, 7H); ^13^C-NMR (CDCl_3_, ppm) *δ* 213.9, 171.5, 141.4, 132.5, 43.5, 35.8, 31.1, 30.8, 29.9, 27.6; Anal. Calcd for C_10_H_12_O_3_: C 66.65; H 6.71. Found: C 66.46; H 6.54.

## 4. Conclusions

In conclusion, the synthesis of enantiomerically pure bicyclic dinitrile **3** and acids **4,** **6** from (+)-(*1S,5S*)-bicyclo[3.3.1]nonane-2,6-dione (**1**) was accomplished and the chiroptical properties of the obtained α,β-unsaturated compounds were studied. The bisignate CE in the CD spectra of difunctional derivatives **3** and **4**, associated with the π→π* transitions apparently originates from the non-exciton-type coupling between the two unsaturated fragments. TD-DFT calculations suggest a substantial through-space interaction (homoconjugation) of π orbitals in **3** and **4**. As a result, the studied molecular structures should be treated as specific delocalized electron systems, where the two unsaturated moieties are conjugated to each other, losing their nature as an independent unsaturated chromophore. The analysis of stereostructure of such fully delocalized conjugated *C_2_*-symmetric monochromophores, which acquire left-handed (M) helicity in *(1S,5S)-***3** and *(1S,5S)-***4**, by the exciton chirality method is therefore not justified. The sign of the CE’s in the CD spectra of **3** and **4**, negative at the longer wavelengths and positive at shorter wavelengths, is decided by inherent dissymmetry (helicity) of the extended monochromophores and is in accordance with a general helicity rule.
